# Relaxations and Hobbies

**Published:** 1913-10-18

**Authors:** 


### RELAXATIONS AND HOBBIES.

(Contributions are Invited.)

### Nature Notes.

"Cock's Nests"—The True Explanation—Nest Architecture—Apparent Desertion—Curious Facts—A Difficult Question.

On a page of Nature Notes recently published I set out certain ideas I had about the nesting habits of the wren. The gist was that for years it had been my belief, because told me in my boyhood and seemingly confirmed by incomplete observations, that wrens built what are called "cock's nests" as shelters for cold nights and that they, more than other birds, are apt to desert their breeding nests if these should be touched before egg-laying has commenced. I added that my own observations had taught me that the wren is at least as tolerant of interference with its nest as any other bird when eggs have been laid, and that certain observations lately made had led me to think I had discovered facts about the nesting of wrens not generally known. Further observations have convinced me that my surmises were correct.

Early in April, if not before, wrens begin to make nests. They work energetically for a few days till the structures assume so far the form of the finished nest that anyone can tell what bird is the architect. At this point often they pause in their work for many days. I have had opportunity to watch closely two such beginnings. In each case I saw one bird only occupied about the nest at the same time. I mean that on no occasion did I ever see two birds at once at the nest. After construction had continued for a few days the nests in each case were abandoned, or seemed to be abandoned, and remained for weeks untouched and, so far as my observations went, unvisited. At this stage the nests, especially the first, which was in some honeysuckle, were typical of the structures I had learnt to call "cock's nests," and which it is said are built only as temporary shelters for cold nights.

In each case, after some weeks, I saw the birds occasionally visit the nests and sometimes add to them till they were completed, or practically completed. After that the birds did not visit them, so far as I know, for a considerable period. During this time the first nest was constantly wet with rain so heavy that the mossy walls were soaked right through. For so long were they left that I thought both nests had been deserted because of my examinations; but the birds returned to full occupation, laid, hatched, and successfully reared young. I especially carefully kept watch on the second nest, because I was on the alert to confirm my former observations. It was in the roof of an old tool-shed that I wished to remove from my garden, but when I found a wren had begun to build in it I ordered the shed to be left and directed that care should be taken to avoid damage to the nest. The building began early in April. In the beginning of July, though it had been to all appearance quite finished early in June, there were no eggs in the nest and the wren was seldom seen near it, though at intervals one or another of my household saw her slip in or out of the shed. About the middle of July I discovered three eggs, and the clutch increased to seven or eight; the bird sat; the young ones left the nest early in August.

My head when I was in the shed would often be only a few inches from the entrance of the nest, yet the bird would remain sitting till I turned my face to her and looked in. As soon as she saw she was being looked at she would slip out and off through a broken pane of glass. I seldom saw the bird with food in her beak for her young, and I never caught her feeding them, but she and her mate must have managed to slip in and out unobserved many times a day, as young birds require frequent feeding, and, though hers would gape always if the nest was gently touched, they did not seem more hungry than other nestlings. As soon as the young left the nest the whole party deserted the garden. I have no doubt they are still amongst the thick hedges, shrubberies, and faggot-piles in the orchards just beyond.

I do not assume that every wren makes a nest so far in advance of its needs as these two I have observed. If that were so it would follow that some must begin to nest in February to get eggs laid in May. What I have observed is that some wrens do begin their nests a long time before they lay in them, continue the work with intervals of idleness, or at any rate with intervals of no work on their homes; complete the nests, leave them for weeks apparently neglected and forgotten, and then suddenly return and use them. These are curious facts worthy of further observation. It is not the habit of any other of the common English birds so far as I know, nor can I think of any facts I have observed that make me think it may be the habit of some of them and overlooked by me. I think it probable that the "cock's nests" of the legends of my boyhood and of many popular natural history books will prove in all cases, if they are carefully watched, to be the beginnings of real nests and that, if left alone, they usually will be found to be completed though not perhaps till several weeks have passed; and that the reason completed wrens' nests are so often thought to have been deserted on account of the bird's jealousy of interfering fingers is that a long time may normally elapse between completion and use. The egg collector, after a few visits, loses patience, concludes because the nest has remained complete but empty for some days that it has been deserted and goes near it no more. A visit a month later might find it full of eggs.

Why does this strange little bird that looks as if it should live in the tropics, or at any rate migrate to a warmer land than England for the winter, elect to remain with us all the year round, and why has it developed this unusual nesting habit?

